# Autoimmune encephalitis: clinical presentation, investigation and treatment

**DOI:** 10.1007/s00415-021-10800-6

**Published:** 2021-09-21

**Authors:** A. Al-Ansari, N. P. Robertson

**Affiliations:** 1 Department of Neurology, University Hospital of Wales, Heath Park, Cardiff, CF14 4XN UK; 2 Department of Neurology, Division of Psychological Medicine and Clinical Neuroscience, Cardiff University, University Hospital of Wales, Heath Park, Cardiff, CF14 4XN UK

Autoimmune encephalitis is an immune-mediated condition traditionally presenting with cognitive decline, seizures, psychiatric symptoms, and movement disorders. Despite an increasing number of implicated antibodies, the diagnosis of autoimmune encephalitis remains challenging because of the clinical overlap with a broad range of other neurological conditions. This month’s journal club explores three papers relating to clinical presentation, investigation and treatment options for refractory autoimmune encephalitis.

The first paper is a cohort study evaluating resemblance in clinical presentation and ancillary testing between autoimmune encephalitis and neurodegenerative syndromes. The second paper is a multicentre retrospective review of patients with autoimmune encephalitis who underwent continuous EEG monitoring and propose the presence of a signature EEG pattern for NMDA encephalitis. The third paper describes the use of a Janus kinase inhibitor in the management of refractory cases of autoimmune encephalitis.

## Autoimmune encephalitis resembling dementia syndromes

Autoimmune encephalitis can mimic neurodegenerative dementia syndromes, which may result in misdiagnosis and delayed immunotherapy. The aim of this study was to evaluate dementia symptoms in confirmed cased of autoimmune encephalitis and identify red flag features for autoimmune encephalitis in middle-aged and elderly patients. In this observational cohort study, patients with anti-leucine-rich glioma-inactivated 1 (LGI1), anti-N-Methyl-D-aspartic acid receptor (NMDA-R), anti-gamma-aminobutyric acid B receptor (GABA-B-R) or anti-contactin-associated protein-like 2 (CASPR-2) encephalitis were included. The authors state that these are the most common antibodies causing autoimmune encephalitis, with cognition frequently affected in these subtypes.

Patients were identified between 1999 and 2019 through the department of neurology of the Erasmus University Centre in Denmark, which is the national referral site for patients with autoimmune encephalitis. Patients diagnosed with autoimmune encephalitis over the age of 45, who fulfilled internationally accepted dementia criteria (2011 NINCDS-ADRDA), and had no prominent seizures early in the disease course, were invited to participate (*n* = 67). Data were obtained regarding the clinical phenotype, diagnostic workup, and where possible, CSF biomarkers relating to neurodegenerative syndromes.

Of the 67 patients included in the study, 42 had anti-LGI1 encephalitis, 13 anti-NMDA-R encephalitis, 8 anti-GABA-B-R encephalitis, and 4 anti-CASPR-2 encephalitis. Patient with CASPR-2 encephalitis were excluded from statistical analysis and described exploratively due to the small number.

98% of 63 (*n* = 62) patients had cognitive deterioration, and 87% (*n* = 55) had behavioural changes. There was a rapidly progressive deterioration of cognitive symptoms in 76% (*n* = 48) of patients. A neurodegenerative syndrome was suspected in 52% (*n* = 33) of cases. Patients with anti-LGI1 and anti-GABA-R demonstrated impairment of visuospatial and executive functions, while patients with anti-NMDA-R encephalitis exhibited impaired language function, behavioural change and movement disorders. 64% (*n* = 40) of patients developed seizures during the disease course. Of those, 28% had developed subtle seizures which were missed in the first weeks of disease onset; the most subtle seizures were seen in anti-LGI-1 encephalitis.

Normal routine CSF results and an absence of mesial temporal lobe abnormality was found in 53% and 54% of patients respectively. CSF was most frequently normal in LGI1 encephalitis (76%, *p* ˂ 0.0001). 14 patients were considered to have a CSF biomarker profile in keeping with Alzheimer’s disease or Creutzfeldt–Jakob disease.

*Comment*. This study highlights the prominence of dementia symptoms in several subtypes of autoimmune encephalitis. Investigation in autoimmune encephalitis can often be normal and CSF biomarkers in autoimmune encephalitis can closely resemble a dementia syndrome. Rapidly progressive cognitive decline, seizures and evidence of neuroinflammation in ancillary testing were identified as ‘red flag’ features for autoimmune encephalitis. Subtle brachio-facial-dystonic seizures were commonly missed in early anti-LGI1 encephalitis. Strengths of this study include the national recruitment of a broad range of encephalitis subtypes, and availability of CSF data. A larger study looking at antibody prevalence and response to immunotherapy in patients with presumed dementia may consolidate these findings.

Bastiaansen et al. (2021) Neur Neurimmunol Nuroinflamm. https://doi.org/10.1212/NXI.0000000000001039

## Continuous EEG findings in autoimmune encephalitis

Seizures are a common clinical feature of autoimmune encephalitis. This study describes the findings of continuous EEG monitoring in autoimmune encephalitis. This was a retrospective review of 400 patients identified through a code search for the diagnosis “encephalitis”. Adult patients who presented to hospital with symptoms consistent with autoimmune encephalitis and underwent at least 6 h of continuous EEG monitoring were recruited. The decision to perform EEG monitoring was made by the treating team. Patients with known central nervous system infection, malignancy, brain injury or known seizure disorder were excluded from the study.

Of 64 patients, 43 had antibody-proven autoimmune encephalitis with subtypes as follows: NMDA (*n* = 17, 27%), voltage-gated potassium channel (VGKC) (*n* = 16, 25%), glutamic acid decarboxylase (GAD) (*N* = 6, 9%) and other (*n* = 4, 6%). The remaining patients were classed as probable antibody-negative autoimmune encephalitis (*n* = 11, 17%), definite antibody-negative limbic encephalitis (*n* = 2, 3%) and Hashimoto’s encephalopathy (*n* = 8, 13%). The diagnosis of antibody-negative autoimmune encephalitis was made using previously published criteria.

EEG reports were reviewed retrospectively and noted for the presence of periodic or rhythmic patterns, focal or generalised seizure activity, seizure type, and the presence of new onset refractory status. EEG patterns were coded according to the American Clinical Neurophysiology Society critical care EEG nomenclature. There were no statistically significant differences between autoimmune encephalitis subtypes with regards to demographics, clinical features, radiographic findings, CSF findings, rates of non-convulsive status or outcomes at discharge.

There was a high incidence (54%) of electrographic seizures in the study group. New onset refractory status epilepticus was seen in 19% of all patients. Generalised rhythmic delta activity (GDRA) was identified significantly more often in anti-NMDA receptor encephalitis (*p* = 0.0001), in fact anti-NMDA receptor encephalitis was the only subtype that demonstrated GRDA plus fast (GDRA + F) subtype (*p* = 0.0001). No specific EEG findings were identified in association with any other antibody causing autoimmune encephalitis. The presence of non-convulsive status conferred the highest risk of a poor outcome. The presence of GDRA did not correlate to a poor outcome in this study.

*Comment*. The authors suggest that GRDA + F is synonymous with the extreme delta brush (EDB) pattern previously reported as characteristic of NMDA encephalitis and conclude that this pattern is a useful biomarker for NMDA encephalitis. Limitations of this study include the small numbers of autoimmune encephalitis subtypes, and unclear definitions of some antibody-negative subtypes. EEG review was also retrospective, and a limited range of EEG outcomes were assessed, and assessment of interrater reliability was not available.

Moise et al. (2021) J Clin Neurophysiol. https://doi.org/10.1097/WNP.0000000000000654

## Tofacitinib treatment for refractory autoimmune encephalitis

Tofacitinib is a Janus kinase inhibitor with effective penetration of the blood–brain barrier, already in use in refractory rheumatoid arthritis and ulcerative colitis. This paper describes outcomes in eight patients with refractory autoimmune neurological syndromes treated with Tofacitinib who did not previously respond to conventional immunotherapy.

A total of eight patients were treated with Tofacitinib. Two patients had NMDA-R antibodies, one had anti-GAD antibodies and one had myelin-oligodendrocyte glycoprotein (MOG) antibodies. Four patients had no detectable antibodies. The clinical syndromes for these patients comprised limbic encephalitis (*n* = 4), rhombencephalitis (*n* = 2), stiff-person syndrome (*n* = 1) and non-convulsive status (*n* = 1). All patients previously had an unclear response to steroids, immunoglobulin therapy, and a range of steroid-sparing agents. Tofacitinib was administered at 5 mg twice daily. Patient outcomes were assessed using a number of assessment tools including the modified Rankin scale.

The treatment efficacy of Tofacitinib was assessed as good in two patients and partial in three patients. There was no response in three patients. The two patients with favourable responses are described. One patient had a chronic meningoencephalitis refractory to steroids, immunoglobulin therapy and steroid-sparing agents, which responded clinically and radiologically to Tofacitinib administered over a month. The second case of non-convulsive status with MOG antibodies refractory to anti-epileptic therapy and immunotherapy responded to Tofacitinib with cessation of epileptiform discharge 16 days post-administration. In the patients with partial response, Tofacitinib stopped the progression of active disease. Tofacitinib was well tolerated in six patients. One patient reported nausea, another had transient neutropenia necessitating withdrawal of Tofacitinib.

*Comment*. This study describes a small case series of patients with a range of refractory neurological syndromes. Overall, Tofacitinib was well tolerated in seven out of eight patients, and six patients demonstrated a partial or good response. This is a promising initial report for the use of JAK inhibitors in a range of refractory autoimmune neurological conditions. Further studies with larger numbers of patients with confirmed antibody-mediated encephalitis would be helpful in determining optimal doses and duration of treatment.

Jang et al. (2021) Epilepsia. https://doi.org/10.1111/epi.16848

## Conclusion

The three papers described in this month’s journal club allow us to improve our understanding of the presentation and management of autoimmune encephalitis, which should be considered early in cases of rapidly progressive dementia with seizures. Furthermore, EEG signatures may be characteristic of autoimmune encephalitis subtypes, with encouraging reports emerging for targeted immune treatment strategies.

